# The Effect of Branding to Promote Healthy Behavior: Reducing Tobacco Use among Youth and Young Adults

**DOI:** 10.3390/ijerph14121517

**Published:** 2017-12-07

**Authors:** Donna Vallone, Marisa Greenberg, Haijun Xiao, Morgane Bennett, Jennifer Cantrell, Jessica Rath, Elizabeth Hair

**Affiliations:** 1 Truth Initiative Schroeder Institute, 900 G Street NW, Fourth Floor, Washington, DC 20001, USA; mgreenberg@truthinitiative.org (M.G.); jxiao@truthinitiative.org (H.X.); mbennett@truthinitiative.org (M.B.); jcantrell@truthinitiative.org (J.C.); jrath@truthinitiative.org (J.R.); ehair@truthinitiative.org (E.H.); 2 Department of Health, Behavior and Society, Johns Hopkins Bloomberg School of Public Health, Baltimore, MD 21205, USA; 3 College of Global Public Health, New York University, New York, NY 10003, USA

**Keywords:** brand equity, health promotion campaign, tobacco, health behavior, branding, smoking

## Abstract

Policy interventions such as public health mass media campaigns disseminate messages in order to improve health-related knowledge, attitudes, beliefs and behaviors at the population level. Only more recently have campaigns that promote health-related behaviors adopted branding, a well-established marketing strategy, to influence how consumers think and feel about a message. This study examines whether positive brand equity for the national truth^®^ campaign is associated with lower likelihood of cigarette use over time using the nationally representative Truth Longitudinal Cohort of youth and young adults, aged 15–21. Logistic regression models were used to examine the relationship between brand equity and the likelihood of reporting past 30-day smoking over a 12-month period. Respondents who reported positive brand equity were significantly less likely to report past 30-day smoking 12 months later (OR = 0.66, *p* < 0.05), controlling for covariates known to influence tobacco use behavior. Findings also translate the effect size difference to a population estimate of more than 300,000 youth and young adults having been prevented from current smoking over the course of a year. Building brand equity is a strategic process for health promotion campaigns, not only to improve message recall and salience but also to influence behavioral outcomes.

## 1. Introduction

Policy interventions such as public health mass media campaigns disseminate messages to help improve health-related knowledge, attitudes, beliefs and behaviors at the population level. Over the past few decades, these types of campaigns have met with varying success [1,2], and their effectiveness has been difficult to measure [3]. Health communication experts urge campaign planners to employ innovative strategies to persuade consumers of the value of a healthy lifestyle in an increasingly crowded marketplace [4]. Only more recently, however, have campaigns that promote health-related behaviors adopted branding, a well-established marketing strategy proven to enhance communication efforts [2,5,6]. Branding aims to reflect a complex system of values and personality by tailoring the language, tone of voice, messaging, product delivery, and all components of visual media to promote and identify the brand [7,8]. Simply put, brands help influence how consumers think and feel about a product, service or message [9]. Ideally, the symbolic representation (i.e., label, sign, or symbol) is designed to activate recall of positive brand characteristics, without necessarily viewing an ad execution [2]. For example, some of the most effective branding efforts have been employed by tobacco manufacturers to help promote smoking [10,11,12,13,14]. Phillip Morris’ use of sophisticated imagery, carefully-designed packaging, and messages from an iconic cowboy, also known as the Marlboro Man, served to quickly identify smoking as behavior linked to freedom, independence, courage and satisfaction. Branding helped shape a generation in which smoking became a socially desirable behavior, and Marlboro became a cigarette brand of choice [2,12]. 

Health promotion campaigns apply traditional branding principles to promote some type of health behavior as a “product” in exchange for consumer health and wellbeing [4]. The desired behavior is not a purchase decision, rather it is a voluntary, health-promoting behavior that the consumer is asked to initiate or maintain [15]. The associations that consumers make with public health brands can help expedite the communication of branded health messages and engagement in healthy behaviors or lifestyles [2]. For health behavior campaigns, branding can establish long-term value, enabling brand affinity to build with the target audience so that they ultimately adopt and sustain healthy behaviors [16].

To assess the impact of branding strategies, marketing researchers have developed a measure called brand equity—a multidimensional construct designed to assess perceptions about a brand. More specifically, brand equity measures typically include a set of positive and negative brand attributes that are linked to a brand name and symbol. The extent to which consumers perceive the brand as positive or negative can serve to either add to or subtract from the value provided by a product, service, or message [17]. While the benefits of having strong, positive brand equity often prompt consumers to purchase a product or service, it can also extend to increasing effective message recall and comprehension [17,18,19,20,21].

Several studies have examined brand equity as a mediating factor associated with health behavior change campaigns [9,22,23,24,25,26]. Branded messages are more likely to be effective by helping to counter messages that promote unhealthy behaviors [12,24]. For example, the Centers for Disease Control and Prevention’s Verb^®^ campaign employed an effective branding strategy to promote daily physical activity among tweens (aged 9–13 years old) to help counter the promotion of fast food that is associated with childhood obesity [9,27]. By building the brand’s equity and maintaining its integrity, VERB could resonate with tweens and make the topic of physical activity interesting to those of varying demographic characteristics and levels of physical capability.

Measures of brand equity can also be employed to assess the impact of campaign exposure, particularly when campaigns disseminate content through various media channels. As communication has evolved across a variety of devices and platforms, consumers are seeing, reading and hearing ad content from numerous products. Gone are the days of advertising through a relatively limited set of standard 30-s or 1-min ad executions rotated in traditional paid outlets like television and radio. Marketing and promotional efforts now abound across multiple digital platforms, including Facebook, Twitter, and YouTube [28]. Digital content, particularly within social media, is often delivered in a variety of formats to bolster the impact of these other media platforms for influencing large groups of people and changing social norms. Traditional assessment of ad awareness in health campaigns [29,30,31,32,33], which is generally based on reports of exposure to standard television and radio messages, may be limited in capturing the prominence and diversity of modern-day message content, delivery channels, and the influence of branding efforts. Alternatively, the concept of brand equity reflects a multi-dimensional construct that requires some level of message exposure, but also reflects an attitudinal perspective about the message. Thus, this measure may be an important alternative to ad awareness in assessing how and to what extent campaign exposure is associated with behavioral outcomes. This study examines whether positive truth^®^ brand equity is associated with lower likelihood of cigarette use over time using a nationally representative, longitudinal cohort of youth and young adults, aged 15–21.

## 2. Materials and Methods

### 2.1. Description of Truth^®^ Campaign

The national mass media campaign, truth, first launched in 2000 with the goal of preventing tobacco use among youth, aged 12–17, using a comprehensive branding strategy. In 2014, truth relaunched a new campaign focused on a slightly older target audience of youth and young adults, aged 15–21, which encourages young people to join a social movement to end smoking. Campaign advertisements and alternative truth-related content are delivered on television channels and in TV shows popular among the target audience, including MTV and Comedy Central, and online through banner ads, online video ads, homepage takeovers, paid promotions on social media sites, branded social media sites, and a branded campaign website. The campaign is delivered to 210 U.S. media markets. Additional details regarding the delivery of the truth campaign have been described elsewhere [29].

### 2.2. Study Sample

This study uses data from the Truth Longitudinal Cohort (TLC), a nationally representative, probability-based sample of youth and young adults. Detailed methods are available elsewhere [34]. Briefly, the TLC baseline sample included approximately 14,000 respondents age 15–21, with follow-up interviews occurring every six months. This study sample reflects data collected at baseline (April–July 2014), wave 3 (July–October 2015)—the first wave that the Brand Equity scale was included, and wave 5 (July–October 2016)—twelve months later. The sample is limited to only those participants with data at the first five waves of the study who reported awareness of the brand logo or symbol at wave 3 (*n* = 4509), without which they could not respond to questions about the brand. To generalize to the national population of 15–21-year-olds as reflected in the 2014 Census, analyses used sample weights adjusted for selection probabilities and non-response [35]. Details regarding TLC methods and response rates are available elsewhere [34]. All study procedures were reviewed and approved for human subject research by Chesapeake Institutional Review Board (IRB; the cognizant IRB for truth initiative).

### 2.3. Measures

***Brand Equity.*** Brand equity was assessed using the truth Brand Equity scale. The detailed measures in this scale were originally adapted from Aaker [21] and construction of the scale has been described elsewhere [22]. The scale includes items related to the perceptions of the truth brand within four constructs: brand loyalty, leadership/popularity, brand personality, and brand awareness. These four constructs were determined based on: (1) a quantitative assessment of audience perceptions of truth advertising messages; (2) a content analysis of truth initiative social media posts on Twitter, Instagram, and Facebook in which the campaign was actively pushing out messages for three 2-week periods of time in September 2014, January 2015, and March 2015; and (3) previous research that indicated these four constructs formed a higher-order brand equity factor (the full Brand Equity scale) that mediated the effects of exposure to the original truth campaign on smoking outcomes [22]. Items used to measure the brand loyalty construct included: (1) I’d like to help truth end smoking in my generation; (2) I’d defend truth on social media if someone were putting it down; (3) I’d follow truth on social media; and (4) I would be part of a movement to end smoking. Leadership/popularity items included: (1) truth is helping my generation end smoking; and (2) truth is for people like me. Brand personality was assessed with the following items: (1) If truth was a person, truth would be...(a) inspired, (b) powerful, (c) in control of their own decisions, (d) independent, (e) honest, and (f) innovative; (2) People that follow truth are just like me; and (3) People that follow truth are like the friends I hang out with. Finally, brand awareness was measured with the item: When you think of truth, you think, (1) fewer and fewer young people today smoke cigarettes; (2) tobacco companies lie; (3) the tobacco industry tried to get young people to smoke other products like hookah; and (4) tobacco company ads are a joke. All items were listed as statements with a 5-point agreement scale, which was coded so that −2 = strongly disagree, −1 = disagree, 0 = neither, 1 = agree and 2 = strongly agree. Weighted responses for each item are included in Table 1. Mean response scores were then calculated for each construct. Based on prior confirmatory factor analysis results, all items loaded satisfactorily to one of the brand equity constructs, and each construct demonstrated adequate model fit [22].

***Smoking Status.*** The outcome of interest in this study was self-reported past 30-day cigarette use at wave 5. This outcome was defined using the following item: “During the past 30 days, on how many days did you smoke cigarettes (even 1 or 2 puffs)”? Those who reported smoking on one or more days were categorized as current smokers, and this variable was modeled as categorical for this analysis (1 = current smoker, 0 = never smoked or had smoked, but not in the past 30 days).

***Intention to Quit Smoking.*** An additional outcome of interest was intention to quit smoking at wave 5. This variable was defined using the following items: “During the past 30 days, on how many days did you smoke cigarettes (even 1 or 2 puffs)”? and “Do you want to completely stop smoking cigarettes”? Only participants who self-reported smoking on one or more days in the past 30 days saw the second item. Those who answered “yes” to the second item were defined as intending to quit smoking cigarettes (coded as 1). Those who answered “no” were categorized as not intending to quit smoking (coded as 0).

***Control Variables.*** Control variables were identified based on existing literature on predictors of tobacco use or predictors of campaign exposure among youth and young adults. Detailed descriptions of each control variable are included in Table 2 and Appendix A. As not all control variables included in the model were measured at wave 3, measures of these variables at participants’ baseline assessment were included.

***Demographics and psychographics*.** Demographics included age (continuous), gender (male, female), race/ethnicity (White, Black, Hispanic, other), and parent education (higher than high school, HS/GED or less). Psychographics included participants’ media use (low/medium/high), school achievement (much better/better than average, average/below average), and sensation seeking.

***Health*.** Several covariates were included to measures cigarette and other tobacco/substance use, participants’ immediate environment, and perceptions of smoking. A Smoking Intensity measure, adapted from prior work examining progression of youth smoking [36,37,38], was used at baseline to account for differences between those who may have smoked only a few times and those who are more regular or daily smokers. The measure included five levels of smoking progression: (1) Closed to smoking; (2) Open to smoking; (3) Low-intensity non-daily smoking; (4) High-intensity non-daily smoking; and (5) Daily smoking. The first two levels classified those who reported no past 30-day cigarette use. The remaining three levels classified participants who reported past 30-day smoking, and was based on a combination of number of days smoked and the number of cigarettes per smoking day (CPD). Other health-related variables, including participants’ past 30-day use of other tobacco products, household e-cigarette and/or combustible use (no one I live with smokes, someone I live with smokes cigarettes/cigars and/or e-cigarettes), and peer cigarette use.

***Anti-Tobacco Attitudes*.** An 18-item multi-dimensional anti-tobacco scale (ATS) was developed to reflect the five attitudinal constructs that exemplify the themes of the truth campaign: anti-smoking imagery, disapproval of social smoking, support for an anti-tobacco social movement, anti-tobacco industry sentiment, and independence. All attitude items were measured before assessing any campaign awareness at baseline. For each of the 18 items, participants were asked to what degree they agree or disagree with each statement (1 = strongly disagree, 2 = disagree, 3 = neither, 4 = agree, and 5 = strongly agree). Scales were created for each of the five attitudinal constructs, and an average score across the five attitudinal scales was calculated. The ATS scored within the excellent range on reliability (α = 0.90) [29].

***Ad Awareness*.** Awareness of truth advertisements was assessed by calculating an index of the self-reported cumulative number of individual ads seen, as well as the estimated frequency of seeing each ad from baseline to wave 3. Awareness of other national anti-tobacco campaigns, such as the Food and Drug Administration’s (FDA) Real Cost Campaign and the Centers for Disease Control and Prevention’s (CDC) Tips from Former Smokers campaign, was assessed at baseline.

***Policy*.** DMA-level smoking prevalence and tobacco-related policy variables, including county-level smoke free air laws, state-level tobacco control expenditures, and state-level cigarette tax, were included based on participants’ residential address at baseline.

### 2.4. Statistical Analysis

Weighted and unweighted frequencies were calculated to examine sample demographics. Logistic regression models were estimated to determine the influence of brand equity at wave 3 on past 30-day cigarette smoking at wave 5, controlling for the variables described above. Many individual-level variables were modeled as categorical for this analysis. Age, brand equity, sensation seeking, peer behavior, the ATS index and all policy covariates were included as continuous variables (see Table 2 for means and standard errors). Analyses were conducted in Stata/SE 15.0 [39]. Svy command was used to incorporate the sampling weight. 

## 3. Results

Weighted and unweighted demographic characteristics of the study sample are summarized in Table 2. The mean age of the overall sample is approximately 18 years old, with over half of the sample reporting race/ethnicity as non-Hispanic white, heterosexual, having parents with higher than a high school diploma or GED education, doing better than average in school, and utilizing media on a moderate level.

Adjusted and unadjusted logistic regression models were used to estimate the likelihood of self-reported past 30-day cigarette smoking as predicted by the Brand Equity scale 12 months later. The results of the unadjusted model indicated that higher levels of brand equity were significantly associated with a lower likelihood of past 30-day smoking 12 months later (OR = 0.51; 95% CI = 0.40–0.65). The results of the adjusted model were consistent with those of the unadjusted model (OR = 0.64; 95% CI = 0.47–0.87). The findings indicate that a one-point increase on the truth Brand Equity scale is associated with a 36% decrease in the likelihood of reporting current smoking.

Higher levels of smoking intensity were also significantly associated with increased odds of past 30-day smoking 12 months later: open to smoking (OR = 3.66; 95% CI = 2.51–5.33); low intensity non-daily smoking (OR = 5.30; 95% CI = 2.90–9.68); high intensity non-daily smoking (OR = 15.91; 95% CI = 8.38–30.20); and daily smoking (OR = 32.61; 95% CI = 14.02–75.89). Several other factors were found to be significantly associated with increased odds of past 30-day smoking, including reporting average or below-average school achievement (OR = 1.84; 95% CI = 1.35–2.53), living with someone who smokes combustibles and/or e-cigarettes (OR = 2.51; 95% CI = 1.80–3.49), and greater peer cigarette use (OR = 1.27; 95% CI = 1.09–1.48) (see Table 3). 

Table 3 presents the results of the adjusted model including brand equity as a predictor of intentions to quit cigarette smoking 12 months later. The results indicate a statistically significant relationship between higher levels of brand equity and greater intentions to quit cigarette smoking over 12 months (OR = 2.31; 95% CI = 1.49–3.58). Moreover, when controlling other variables, those reporting higher levels of brand equity were more than two times more likely to report intentions to quit cigarette smoking 12 months later (OR = 2.35; 95% CI = 1.22–4.54). 

Lower levels of smoking intensity were significantly associated with increased intentions to quit smoking: open to smoking (OR = 0.37; 95% CI = 0.16–0.85) and low intensity, non-daily smoking (OR = 0.28; 95% CI = 0.09–0.85). Both low and high ad awareness were significantly associated with higher odds of intending to quit smoking cigarettes in the next year. Those who reported higher levels of ad awareness had greater intentions to quit cigarette smoking compared with those who reported lower levels of ad awareness (low ad awareness: OR = 2.26; 95% CI = 1.18–4.34; high ad awareness: OR = 2.99; 95% CI = 1.02–8.73). 

## 4. Discussion

The study findings demonstrate a significant relationship between truth brand equity and smoking behavior, whereby increasing levels of positive brand equity are associated with both a lower likelihood of reporting past 30-day smoking and greater intentions to quit smoking one year later. This significant finding represents an effect over and above ad awareness as well as key individual, social, and environmental factors known to impact tobacco use behavior. The results highlight the importance of employing consumer-centric branding strategies to help prompt health-promotion message engagement and promote behavior change.

The results from this study also provide estimates of the population impact of the truth campaign. Specifically, we determined the probability of being a current smoker when setting the brand equity value to neutral or 0 and the predicted probability of being a current smoker based on the model estimates. A value of 0 reflects neither a positive nor negative response to brand attributes, when the population impact is assumed to be equivalent to there being no campaign in the market. The estimates indicate that the probability of current smoking is 15.7% at neutral brand equity, or 1.5 percentage points higher than the model-based predicted probability at 14.2%. Applying these predictions to age-specific population numbers from the 2014 Census and incorporating our brand awareness levels, the findings indicate that exposure to the truth campaign was associated with preventing an estimated 301,930 U.S. youths aged 15–21 years from becoming current smokers during 2015–2016. Given the recent increases in tobacco use initiation among young adults [14,38,40], national, population-based health branding strategies are even more important in helping to accelerate the decline in youth and young adult tobacco use, particularly in states and communities without strong tobacco control implementation. 

Brand equity, a relatively new measure within the health-promotion domain, is used here as a measure of message exposure and sentiment. Although cumulative awareness of advertising messages has been used as a predictor of campaign impact, ad awareness measures can be limited since they largely rely on self-reported recall of specific ad executions and frequency of exposure [29]. Given the significant changes in the ways messages are disseminated and individuals interact with brands in the new communications environment, brand equity may serve as an alternative measure of campaign exposure and message impact, particularly for a campaign which heavily focuses on engaging young people through digital and social media which employs integrated content delivery in addition to traditional advertisements. 

Building brand equity as a tool to increase campaign impact is critical, given the challenges of aligning positive health behaviors with consumers’ interests or desires [2,41]. For example, youth and young adults often reject the value proposition of healthy eating, given the advantages of cost, time, and effort associated with junk food [42]. Branding can help make healthy behaviors more personally compelling and culturally relevant for a target audience. Comprehensive studies of audience preferences and culture helps to create compelling brands that reflect the passion points of the intended audience, and which are tied to timely and culturally relevant trends. In the last decade, tobacco use patterns have changed, the communications landscape has significantly shifted, and a new generation of youths and young adults espouse different attitudes, beliefs and behaviors. Branding strategies, like those used by truth, must evolve to stay relevant with a target audience. Findings from this study highlight the need for health-related messages to be culturally relevant, particularly those focused on shaping the health behaviors of youth and young adults. 

### Study Strengths and Limitations

This study is strengthened by the study sample—a large, nationally representative, longitudinal sample—which allows for stronger generalizability of the study findings. Additionally, the longitudinal nature of the study, whereby brand equity was measured at an earlier time point than the outcome, allows for greater confidence in the direction of the causal chain. Finally, this study used a measure of brand equity that has been validated specifically as it relates to the truth^®^ brand [22], strengthening the reliability and validity of the measure.

Despite the study’s strengths, there are several weaknesses that should be noted. Measures of tobacco use were self-reported, and are therefore subject to biases inherent in this type of measure, such as social desirability bias. However, studies have shown that the validity of reported tobacco use is quite high [43]. Additionally, while the study did include a variety of individual, social, and environmental covariates, the lack of randomization in the study design prevents the researchers from ruling out other possible confounders. While randomizing a mass media intervention to treatment and control media markets may be an ideal way to help eliminate potential confounders, this type of research design is also compromised given the digital dissemination of media messages across geographic media market boundaries.

## 5. Conclusions

The new ways in which individuals learn about and engage with information, ideas and other individuals online and offline demands creative and responsive strategies from marketers and communications experts. Branding, while commonplace in product marketing and advertising, has not been as widely used in the health communications field. Further, campaign evaluation has not kept pace with the new ways health communications marketing is reaching audiences and catalyzing new forms of audience engagement with health brands and messages. This paper utilizes a relatively new measure of campaign impact [22,24] to evaluate the effect of a tobacco prevention campaign on youth and young adult audiences, finding significant reductions in smoking for those with higher brand equity over and above ad awareness. Building brand equity for health-promotion campaigns can serve as a catalyst to improve not only message recall and salience, but can also influence behavioral outcomes. Given the challenges of promoting healthy choices among youth and young adults, efforts to carefully design a culturally relevant branded health-promotion campaign can help shape the next generation in order to significantly improve population health.

## Figures and Tables

**Table 1:** Weighted responses to individual Brand Equity scale items (*n* = 4509).

Construct	Individual Brand Equity Items	Agree/Strongly Agree		M	SE	SD
	How Much Do You Agree or Disagree with the Following?	*n*	%			
**Brand Loyalty**	I’d like to help truth end smoking in my generation	1936	43.0%	0.37	0.01	0.87
	I’d defend truth on social media if someone were putting it down	1680	37.4%	0.22	0.01	0.93
	I’d follow truth on social media	1494	33.2%	0.07	0.01	0.99
	I would be part of a movement to end smoking.	2314	51.3%	0.52	0.01	0.94
**Leadership/popularity**	Truth is helping my generation end smoking.	2558	56.8%	0.57	0.01	0.84
	Truth is for people like me	1726	38.4%	0.25	0.01	0.91
**Brand Personality**	**How much do you agree or disagree with the following? Truth is….**					
	Inspired	3284	73.1%	0.92	0.01	0.84
	Powerful	3060	68.1%	0.84	0.01	0.90
	In control of their own decisions	3495	77.8%	1.02	0.01	0.83
	Independent	3280	73.0%	0.91	0.01	0.87
	Honest	3434	76.4%	0.99	0.01	0.85
	Innovative	3010	67.0%	0.80	0.01	0.88
	People that follow truth are just like me	1160	25.8%	0.07	0.01	0.85
	People that follow truth are like the friends I hang out with	1229	27.3%	0.08	0.01	0.86
**Brand Awareness**	**When you think of truth, you think…?**					
	Fewer and fewer young people today smoke cigarettes	2568	57.1%	0.49	0.01	0.96
	Tobacco companies lie	3227	71.8%	0.89	0.01	0.89
	The tobacco industry tries to get young people to smoke other products like hookah	2471	54.9%	0.54	0.01	1.00
	Tobacco company ads are a joke	2305	51.2%	0.50	0.01	0.96

**Table 2:** Unweighted and weighted demographic/psychographic, health, ad awareness, and policy characteristics of study sample (*n* = 4509).

	**Unweighted %**	**Weighted % (*n*)**
**Gender**		
Male (*n* = 1826)	40.5%	48.0%
Female (*n* = 2683)	59.5%	52.0%
**Race/Ethnicity**		
White, Non-Hispanic (*n* = 2855)	63.8%	52.4%
Black/African American, Non-Hispanic (*n* = 495)	11.1%	16.5%
Hispanic (652)	14.6%	23.6%
Other, Non-Hispanic (*n* = 471)	10.5%	7.5%
**School Achievement**		
Much better/better than average (*n* = 3416)	75.8%	70.3%
Average/below/much worse than average (*n* = 1092)	24.2%	29.7%
**Parent Education**		
Any education higher than high school/GED (*n* = 3811)	85.3%	79.5%
High school/GED or less (*n* = 656)	14.7%	20.5%
**Combined Media Use-TV, Mobile, Computer & Social Media**		
Low—<240 min/day (media) & <1 h/week (social) (*n* = 406)	9.0%	8.2%
Medium—240–599 min/day (media) or 1–3 h/week (social) (*n* = 2315)	51.3%	48.3%
High—600+ min/day (media) or <3+ h/week (social) (*n* = 1788)	39.7%	43.5%
**Cigarette Smoking Intensity**		
Non smoker, closed to smoking (*n* = 3032)	67.2%	65.2%
Non-smoker, open to smoking (*n* = 1078)	23.9%	24.5%
Low intensity smoker, non-daily user (*n* = 161)	3.6%	3.4%
High intensity smoker, non-daily user (*n* = 129)	2.9%	3.6%
Daily smoker (*n* = 109)	2.4%	3.3%
**Past 30-day Use of Other Tobacco Products**		
No (*n* = 3789)	84.0%	82.9%
Yes (*n* = 720)	16.0%	17.1%
**Household E-Cigarette and Combustible Tobacco Use**		
No one I live with smokes (*n* = 3449)	76.5%	75.2%
Cigarettes/Cigars and/or E-cigarettes (*n* = 1060)	23.5%	24.8%
**truth Ad Awareness**		
No awareness (0) (*n* = 1711)	37.9%	37.7%
Low awareness (1–7) (*n* = 2281)	50.6%	49.3%
High awareness (8–28) (*n* = 517)	11.5%	13.0%
	**Unweighted Mean (SE)**	**Weighted Mean (SE)**
**Age (15–21)**		
Mean (years) (SE)	18.2 (0.03)	17.8 (0.06)
**Sensation Seeking Index (1–5)**		
Mean (SE)	2.99 (0.01)	2.96 (0.02)
**Peer Cigarette Use (0–4)**		
Mean (SE)	0.53 (0.01)	0.64 (0.02)
**ATS Index (1–5)**		
Mean (SE)	3.65 (0.01)	3.64 (0.01)
**DMA-Level Smoking Prevalence (10.3–29.6%)**		
Mean (SE)	20.3% (0.05%)	20.0% (0.07%)
**County-Level Smoke-Free Indoor Air Law Index (0–6)**		
Mean (SE)	4.72 (0.03)	4.51 (0.04)
**2013 State-Level Per Capita Tobacco Control Funding ($0.28–$16.72)**		
Mean (dollars) (SE)	$1.75 (0.02)	$1.81 (0.04)
**State-Level Cigarette Tax ($0.17–$4.35)**		
Mean (dollars) (SE)	$1.66 (0.02)	$1.57 (0.02)
**Brand Equity index (−2–2)**		
Mean (SE)	0.50 (0.01)	0.50 (0.01)

**Table 3:** Logistic regression models: Past 30-day smoking and Intention to quit smoking at wave 5.

Variable	Past 30-Day Smoking at Wave 5		Intentions to Quit Smoking at Wave 5	
	Adjusted OR	95% Confidence Interval	Adjusted OR	95% Confidence Interval
**Brand Equity**	0.64 *	**(0.47–0.87)**	2.35 *	**(1.22–4.54)**
**Age**	1.06	(0.99–1.14)	1.09	(0.94–1.27)
**Gender: Female** Ref: Males	0.86	(0.63–1.18)	0.97	(0.53–1.79)
**Race: Black** Ref: White, Non-Hispanic	1.45	(0.91–2.34)	2.26	(0.76–6.74)
**Race: Hispanic** Ref: White, Non-Hispanic	1.08	(0.69–1.68)	0.89	(0.41–1.94)
**Race: Other** Ref: White, Non-Hispanic	0.99	(0.55–1.77)	0.37	(0.12–1.09)
**School Achievement: Average or below** Ref: Much better/better than average	1.85 **	**(1.35–2.53)**	0.97	(0.54–1.75)
**Parent Education: HS/GED or less** Ref: Any education higher than high school/GED	1.26	(0.84–1.90)	1.61	(0.80–3.28)
**Combined Media use: Medium** Ref: Low	1.12	(0.63 –2.02)	2.48	(0.64–9.63)
**Combined Media use: High** Ref: Low	0.95	(0.53–1.73)	1.59	(0.40–6.32)
**Non-smoker, open to smoking** Ref: Non smoker, closed to smoking	3.66 **	**(2.51–5.33)**	0.37 *	**(0.16–0.85)**
**Low intensity smoker, non-daily user** Ref: Non smoker, closed to smoking	5.30 **	**(2.90–9.68)**	0.28 *	**(0.09–0.85)**
**High intensity smoker, non-daily user** Ref: Non smoker, closed to smoking	15.91 **	**(8.38–30.20)**	0.89	(0.33–2.43)
**Daily smoker** Ref: Non smoker, closed to smoking	32.62 **	**(14.02–75.89)**	1.69	(0.57–5.07)
**Past 30-day use of other tobacco products** Ref: No past 30-day use	1.44	(0.99–2.10)	1.26	(0.68–2.36)
**Someone I live with smokes Cigarettes/Cigars and/or E-cigarettes** Ref: No one I live with smokes	2.51 **	**(1.80–3.49)**	0.85	(0.47–1.53)
**Peer Cigarette Use**	1.27 **	**(1.09–1.48)**	0.25	(0.74–1.21)
**Sensation Seeking Index**	1.06	(0.85–1.32)	1.12	(0.78–1.63)
**Anti-Tobacco Sentiment Index**	0.99	(0.71–1.37)	1.51	(0.90–2.55)
**DMA-level Smoking Prevalence**	1.03	(0.98–1.08)	0.93	(0.84–1.04)
**County-level Smoke Free Air Law Index**	1.03	(0.94–1.14)	0.90	(0.76–1.07)
**State-Level Tobacco Control Expenditure**	0.94	(0.86–1.03)	0.93	(0.78–1.11)
**State-Level Cigarette Tax in Dollars**	0.85 *	**(0.71–1.02)**	1.39	(0.99–1.96)
**truth Ad Awareness: Low** Ref: No awareness	1.16	(0.81–1.68)	2.26 *	**(1.18–4.34)**
**truth Ad Awareness: High** Ref: No awareness	1.14	(0.65–2.02)	2.99 *	**(1.02–8.73)**

* *p* < 0.05, ** *p* < 0.01. Models also controlled for awareness of other anti-tobacco media campaigns.

## Appendix A. Description of Survey Items for Control Variables

- *Combined Media Use* was measured at baseline with the items: The number of hours per week spent on media (TV, computer, mobile combined) and social media.
- *School Achievement* measured at baseline with the item: How well would you say you have done in school?
- *Sensation Seeking* was measured at baseline by calculating the average of the following items: (1) I would like to explore strange places, (2) I would like to take off on a trip with no pre-planned routes or timetables, (3) I like to do frightening things, (4) I like wild parties, (5) I like new and exciting experiences, even if I have to break the rules, (6) I get restless when I spend too much time at home, (7) I prefer friends who are exciting and unpredictable, (8) I would like to try parachute-jumping. Response options 1 = strong disagree, 2 = disagree, 3 = neither, 4 = agree, 5 = strong agree.
- *Cigarette Smoking Intensity* was measured at baseline and includes five levels of smoking progression: (1) Closed to smoking; (2) Open to smoking; (3) Low intensity non-daily smoking; (4) High intensity non-daily smoking; and (5) Daily smoking. The first two levels classified those who reported no past 30-day cigarette use. Classification was based on whether they were open to smoking using responses to two questions: “*Do you think you will smoke a cigarette (even 1 or 2 puffs) in the next year?*” and *“Think about the future, if one of your best friends offered you a cigarette (even 1 or 2 puffs) in the coming year, would you smoke it?”*. Level 1, closed to smoking, consisted of those reporting “*definitely not*” to both intention-to-smoke questions. Level 2, open to smoking, consisted of those reporting *“probably not”, “probably yes”,* or *“definitely yes”* to one or both intention-to-smoke questions.
- *Past 30-day Use of Other Tobacco* Products was assessed at baseline. Participants were asked to report past 30-day use of large cigars/little cigars/cigarillos, hookah, pipe, chewing tobacco, snus, e-cigarettes, and e-hookah/e-cigars/vape pens/hookah pens/vape pipes. Responses were dichotomized into 1 = past 30-day use of any tobacco product and 0 = no past 30-day use of any tobacco product.
- *Household Smoking* was measured at baseline with the item: Does anyone you live with currently smoke any of the following tobacco products on some days or every day? With response options: Cigarettes, large cigar/little cigar/cigarillos, hookah, e-cigarettes, e-hookah/e-cigars/vape pens/hookah pens/vape pipes. Responses were dichotomized into 1 = someone in household smokes at least one tobacco products and 0=no one in the household smokes tobacco products.
- *Anti-Tobacco Scale* was measured at baseline and includes the average of five attitudinal constructs: (1) anti-smoking imagery, (2) disapproval of social smoking, (3) support for an anti-tobacco social movement, (4) anti-tobacco industry sentiment, and (5) independence. Each attitudinal construct was constructed by taking the average response of 2–5 survey items that make up each construct. Attitudes related to anti-smoking imagery included the following items: (1) I would never hook up with a smoker; (2) Celebrities who smoke set a bad example; (3) When people post pictures of themselves smoking, they’re encouraging others to smoke; and (4) If I smoke, I will lose respect from others my age. Disapproval of social smoking included: (1) It’s okay to smoke socially when I’m out with my friends; (2) If you only smoke when out with friends, you are not a real smoker; (3) Bumming a cigarette is a great way to start a conversation with someone; (4) People look cool when they smoke; and (5) It’s not a big deal if my friends smoke. Support for an anti-tobacco social movement included: (1) I want my generation to be known as the one that ends smoking; (2) I would be a part of a movement to end smoking; and (3) Taking a stand against smoking is important to me. Anti-tobacco industry sentiment included: (1) I would like to see tobacco companies go out of business; (2) Tobacco companies make me angry; (3) Tobacco companies try to get young people to start smoking; and (4) Tobacco companies lie. Lastly, independence from tobacco included: (1) Not smoking tobacco is a way to show my independence and (2) Not smoking tobacco makes me feel powerful. Items in the disapproval of social smoking construct were reverse coded as 1 = strongly agree, 2 = agree, 3 = neither, 4 = disagree, and 5 = strongly disagree for ease of interpretation. Each of the five altitudinal construct scales were within the acceptable or good range on reliability (anti-smoking imagery: α = 0.71; anti-social smoking sentiment: α = 0.82; anti-tobacco social movement: α = 0.82; anti-tobacco industry: α = 0.80; independence: α = 0.75).
- *Truth^®^ Ad Awareness* was assessed by calculating an index of the self-reported cumulative number of individual ads seen as well as the estimated frequency of seeing each ad across waves. Participants were shown four screenshots for each truth ad and asked, “Have you seen this ad in the last 6 months?” Those who responded, “yes” were then asked how frequently they saw the ad, with response options 1 = rarely, 2 = sometimes, 3 = often, and 4 = very often. Those who have not seen the ad were recoded as 0 = not aware. Awareness of a total of 12 truth advertisements was asked over the two data collection waves. A cumulative ad exposure index was defined as the sum of recall frequency across all 12 ads (total range from 0–48). Low ad exposure was defined as a score of 1–15 (equivalent to seeing less than four ads very often), and high ad exposure defined as a score of 16–48 (equivalent to seeing at least four ads very often).
- *Designated Market Area (DMA)-Level Smoking Prevalence.* DMA is the standard geographical unit used to measure mass media. Measured based on participants’ residential address at baseline.
- *Smoke-free Indoor Air Law* index was based on the smoke-free air coverage for workplaces, restaurants, and bars. Each of the three locales’ smoke-free air coverage was classified as having either: 0 = No coverage, 1 = Qualified/Some coverage, or 2 = 100% smoke-free coverage. Scores were summed across the three locales to generate an index ranging from 0–6. Measured based on participants’ residential address at baseline [44].
- *State-Level per Capita Tobacco Control Funding.* Based on participants’ residential address at baseline [45].
- *State-Level Cigarette Tax*. Based on participants’ residential address at baseline [46].
